# Nitrogen-doped porous carbon monoliths from polyacrylonitrile (PAN) and carbon nanotubes as electrodes for supercapacitors

**DOI:** 10.1038/srep40259

**Published:** 2017-01-11

**Authors:** Yanqing Wang, Bunshi Fugetsu, Zhipeng Wang, Wei Gong, Ichiro Sakata, Shingo Morimoto, Yoshio Hashimoto, Morinobu Endo, Mildred Dresselhaus, Mauricio Terrones

**Affiliations:** 1 School of Engineering, The University of Tokyo, Bunkyo-ku, Tokyo 113-0032, Japan; 2 Policy Alternative Research Institute, The University of Tokyo, Bunkyo-ku, Tokyo 113-0032, Japan; 3 Institute of Carbon Science and Technology, Shinshu University, 4-17-1 Wakasato, Nagano 380-8553, Japan; 4 Research Laboratory of Electronics, Department of Electrical Engineering and Computer Science, Department of Physics, Massachusetts Institute of Technology, 77 Massachusetts Avenue, Cambridge, MA 02139-4307, USA; 5 Department of Physics, Department of Chemistry, Department of Materials Science and Engineering and Center for 2-Dimensional and Layered Materials, The Pennsylvania State University, University Park, PA 16802, USA

## Abstract

Nitrogen-doped porous activated carbon monoliths (NDP-ACMs) have long been the most desirable materials for supercapacitors. Unique to the conventional template based Lewis acid/base activation methods, herein, we report on a simple yet practicable novel approach to production of the three-dimensional NDP-ACMs (3D-NDP-ACMs). Polyacrylonitrile (PAN) contained carbon nanotubes (CNTs), being pre-dispersed into a tubular level of dispersions, were used as the starting material and the 3D-NDP-ACMs were obtained via a template-free process. First, a continuous mesoporous PAN/CNT based 3D monolith was established by using a template-free temperature-induced phase separation (TTPS). Second, a nitrogen-doped 3D-ACM with a surface area of 613.8 m^2^/g and a pore volume 0.366 cm^3^/g was obtained. A typical supercapacitor with our 3D-NDP-ACMs as the functioning electrodes gave a specific capacitance stabilized at 216 F/g even after 3000 cycles, demonstrating the advantageous performance of the PAN/CNT based 3D-NDP-ACMs.

Responding to the increasing demands for the clean energy-related technologies, electrochemical capacitors (ECs) are considered as one of the most promising energy storage technologies for their applications in electrical vehicles, portable/mobile electronics, or other storage systems based on sources of solar cells and windmills. They have energy densities much higher than those of conventional capacitors and possess much higher power densities than batteries^1^,^2^. Nanocarbon is therefore a promising electrode materials candidate for supercapacitors. Other two candidates, such as transition metal oxides and conducting polymers, suffer from several key drawbacks of their own. The abundance of carbon sources for nanocarbons and the facile processes of their modification have led to the mass fabrication of cheap and high-performance electrodes based on carbon^3^,^4^,^5^,^6^.

Nevertheless, fundamental improvements of carbon electrode materials are needed. Energy storage and power output in carbon-based supercapacitors intensively depend on charge uptake and ion diffusion capability on the carbon/electrolyte interface, which relates to surface chemistry and the electronic structure of porous carbons^6^,^7^. Needed enhancements to the characteristics of porous carbons for this application can be ascribed mainly to improve their pore textures and electronic conductivity^8^.

Since the hierarchical structures of porous carbon can greatly facilitate the ion migration of electrolytes in electrode materials, much effort has been focused on producing carbon materials with graded pores^9^,^10^,^11^. The design and incorporation of three-dimensional (3D) hierarchical porous carbon materials, especially the newly-developed heteroatom-enriched carbon monoliths with interconnected (both macroporous and mesoporous) networks and in one piece, can lead to a significant enhancement in the electrochemical charge/discharge process by improving the hydrophilicity between the interfaces of electrolytes and electrode materials, and also by improving the poor ionic transport of electrolytes in electrode materials.

In this communication, a new kind of carbon/carbon monolith presenting pseudocapacitance properties and high electrical conductivity is reported, obtained by a two-step route from polyacrylonitrile (PAN) and multi-walled carbon nanotubes (CNTs). PAN has here been selectived to produce an electrochemically-active carbon-network matrix containing in-frame incorporated nitrogen, because of its high carbonization yield and high controllable residual nitrogen content in the activated carbon monoliths (ACMs). CNTs were used as a minor electrode component to improve the electrical conductivity and mechanical properties, offering additional porosities in the composite. Firstly, an advanced template-free temperature-induced phase separation (TTPS) method was introduced to prepare both the 3D hierarchical PAN entire monolith and the PANCNT composited monoliths with specially designed morphologies and macro-mesopores. The shapes and volume sizes of the monoliths can be easily tailored by the various vessels that are used in the TTPS process. The subsequent heat treatment process maintains the macropores of the PAN and PANCNT monoliths. Many mesopores could be formed due to the release of a significant amount of gases during PAN’s thermal decomposition. The CNTs-constructed monolith frameworks provide additional pores through a synergetic effect between PAN and CNTs. The as-prepared carbon monoliths are then used in the three-electrode capacitor, demonstrating interesting pseudocapacitance properties related to the presence of the nitrogen species.

## Results

**Figure 1:**
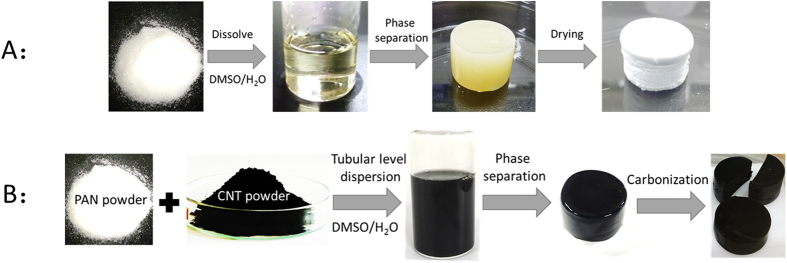
Preparation of typical three-dimensional (3D) hierarchical PAN (**A**) and PANCNT (**B**) monoliths.

Various shapes and volume sizes of the PAN and PANCNT monoliths could be synthesized through the TTPS method, as shown in the supporting information (see Figure S1). Figure 1 demonstrates the preparation process of the typical three-dimensional (3D) hierarchical PAN and PANCNT monoliths and Figure S1 shows the photos of 3D hierarchical, film-like, and plate-like PAN and PANCNT products with different sizes.

**Figure 2:**
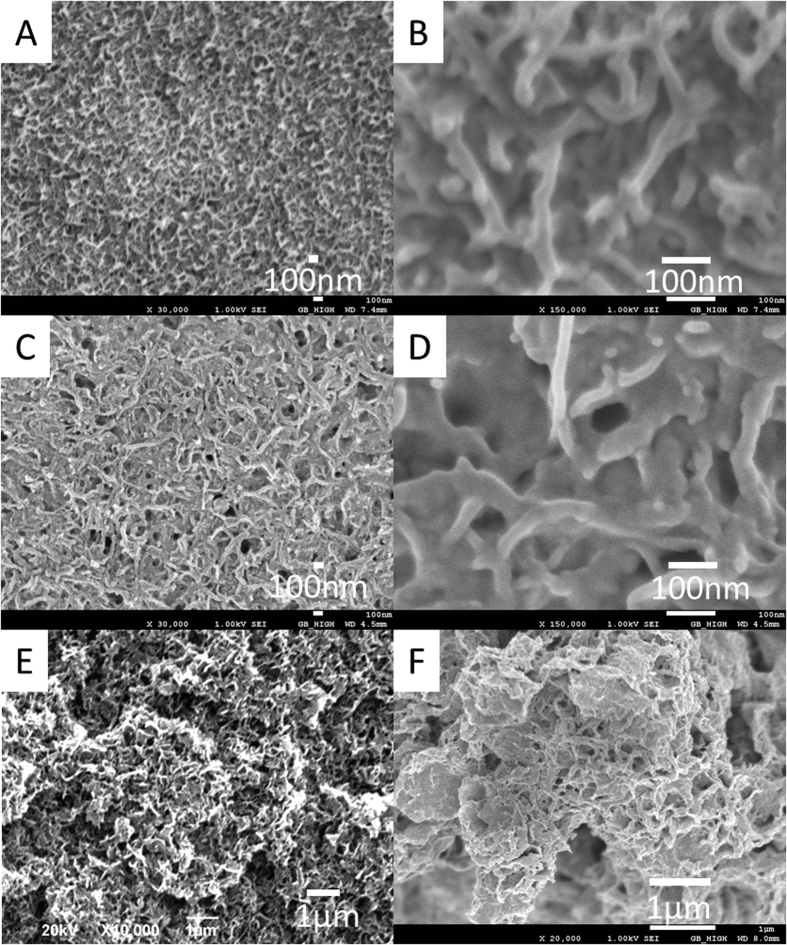
SEM images of pure PAN monoliths (**A**,**B**), PANCNT composite monoliths with the contents of CNTs ranging from 1.0wt. % (**E**), to 6.5wt. % (**C**,**D**) and to 8.5wt. % (**F**).

As indicated in Fig. 2, the morphological properties of PAN and PANCNT monoliths can be obtained by scanning electron microscopy (SEM). Both PAN (Fig. 2A,B) and PANCNT (Fig. 2C,D, 6.5(wt.) % CNTs) monoliths show a 3D network structure, with an identical skeleton size of ~30 nm. By decreasing the content of CNTs (Fig. 2E, to 1.0(wt.) % CNTs) and increasing the content of CNTs (Fig. 2F, to 8.5(wt.) % CNTs) in the PANCNT composite monoliths, the formation of a continuous network structure in the monoliths is hindered. By further increasing the content of CNTs, phase separation failed because of the monolith’s high viscosity (see Figure S2).

**Figure 3:**
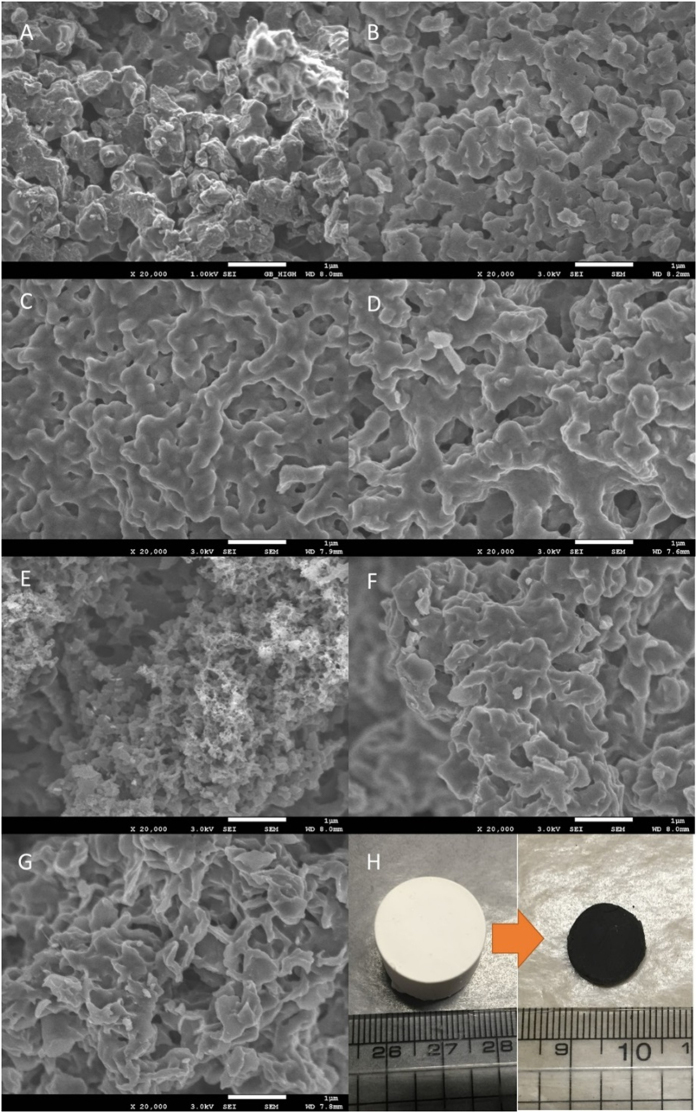
SEM images of carbon monoliths: (**A**) ACM-1, (**B**) ACM-2, (**C**) ACM-3, (**D**) ACM-4, (**E**) ACM-5, (**F**) ACMCNT-6, (**G**) ACMCNT-7, and (**H**) typical photos of the transformation of the PAN monolith into an ACM showing no drastic volume shrinkage or crack formation during heat treatment at 1000 °C for 2 h.

During the heat treatment process of PAN and PANCNT monoliths, the shapes and special morphologies of the monoliths can be maintained without any obvious cracks (see Fig. 3). CNTs incorporated into ACMs show enhanced crushing resistance in the composite, indicating that CNTs act as a reinforcing backbone and the CNTs construct 3D network types of frameworks preventing drastic dimensional changes or cracks in the formation of ACMs.

Morphologies, porosity and nitrogen content of the obtained ACMs can be easily tailored by controlling the heat treatment temperature. In addition, the hold time of the heat treatment has a profound effect on the nitrogen contents of the samples. By observing the cross sections of the ACMs, we found that the skeleton size of the carbon networks (~300 nm) change very little during 700–900 °C heat treatment, but they decrease in size to ~20 nm when carbonized at 1000 °C (see Fig. 3). At 600 °C or lower, deficient carbonization affects the formation of carbon frameworks in the ACMs. A certain amount of volume shrinkage (diameter ~40%, height ~50%) and residual content loss (20~50% in weight) can be observed through carbonization.

Raman scattering spectroscopy is used to investigate the local structures of the carbon monoliths. Three main peaks (D, G, 2D peaks) can be found in the Raman spectra (see Fig. S3), centered at around 1350, 1580 and 2800 cm^−1^, respectively. The D peak corresponds to the A_1g_ mode caused by the structural defects that are associated with a disordered carbon structure (D-band). The G peak, corresponds to the E_2g_ mode that is associated with the C=C stretching in the graphitic carbon (G-band)^12^. In the case of CNTs-backboned carbon monoliths, the ratio of the intensity of the D-band to G-band (*I*_*D*_/*I*_*G*_) are 2.69 (ACMCNT-6) and 2.38 (ACMCNT-7), respectively, which are both smaller than that of the single PAN derived carbon monoliths: ACM-1 (3.91), ACM-2 (3.46), ACM-3 (3.54), ACM-4 (3.32), ACM-5 (3.36) (Table S1). These results indicate that PAN derived carbon monoliths exhibit a relatively more disordered carbon structure compared to that of ACMCNT composite monoliths^13^,^14^.

Electrical conductivity of the PAN and PANCNT derived carbon monoliths are investigated by 4-point probe conductivity measurements at room temperature. As seen in Table S2, the electrical conductivity of the PAN derived carbon monoliths increased by increasing the temperature. The electrical conductivity values of the carbon monoliths from 600–1000 °C are 396.3 × 10^3^ Ω/sq (ACM-1), 1.655 × 10^3^ Ω/sq (ACM-2), 72.9 Ω/sq (ACM-3), 33.7 Ω/sq (ACM-4), 13.4 Ω/sq (ACM-5), respectively. PANCNT derived carbon monoliths demonstrate a better electrical conductivity, 4.67 × 10^3^ Ω/sq for ACMCNT-6 and 6.3 Ω/sq for ACMCNT-7, which indicates that an enhanced electrical conducting network is formed by the backboned-CNTs^15^.

**Figure 4:**
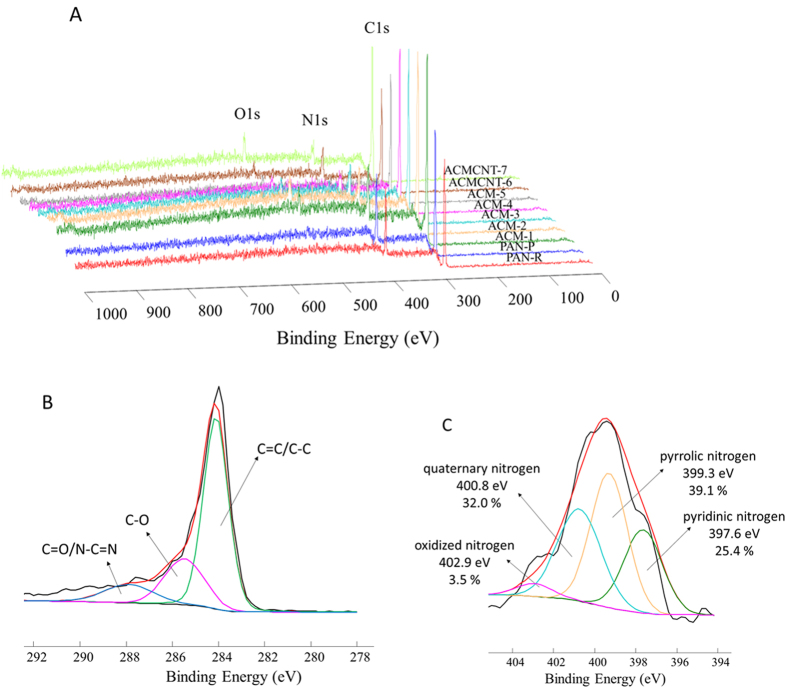
Wide scan XPS spectra of the PAN monolith (PAN-R), the stabilized PAN monolith (PAN-P), the ACM carbon monoliths from 600 to 1000 °C (denoted as ACM-1, ACM-2, ACM-3, ACM-4, ACM-5, respectively), and the ACMCNT carbon monoliths at 600 °C (ACMCNT-6) and 900 °C (ACMCNT-7) (**A**). High-resolution spectra for C 1 s (**B**) and for N 1 s (**C**) of ACM-5.

The wide range XPS spectra in Fig. 4 confirmed three special peaks located at 284.8 (C 1 s), 399.5 (N 1 s) and 533.4 (O 1 s) eV, respectively, indicating that all ACM samples were composed of C, N and O elements. The C content increases and the N content decreases gradually by increasing the carbonization temperature and the carbonization hold time. As shown in Table S2, the C content increased significantly from 79.2% (ACM-1, 600 °C) to 93.7% (ACM-5, 1000 °C). Reversely, while with an increase of the hold time from 1 h to 2 h, regarding the nitrogen content of the ACM monoliths and ACMCNT monoliths, we found that the nitrogen content decreases slightly with the increase of hold time for these samples. For example, the N contents of ACM-8, ACM-9, ACMCNT-10, and ACMCNT-11 (hold time 1 h) are 18.3%, 10.8%, 16.5%, and 10.9 %, respectively, which are larger than their corresponding comparison samples (17.2% for ACM-1, 9.2% for ACM-4, 15.8% for ACMCNT-6 and 9.0% for ACMCNT-7) at the hold time of 2 h. The O content appears in the oxidation step and changes negligibly during the increase of the treatment temperature. As further confirmed in Fig. 4(B), the C 1 s spectrum of ACM-5 has three components attributable to carbon atoms and each are involved in different functional groups: the aromatic C=C/C-C (curves in green color), C-O (curves in magenta color), and carbonyl (C=O and N-C=N) (curves in blue color) species, centered at 284.1, 285.4, and 287.8 eV, respectively^16^,^17^. To understand the role of nitrogen functionalities in their electrochemical performance, it is necessary to clarify the types of nitrogen-containing species introduced onto/into the graphitic carbons. As indicated in Fig. 4(C), the N 1 s spectrum of ACM-5 can be divided into four peaks, including the pyridinic nitrogen (397.6 eV), the pentagonal pyrrolic nitrogen (399.3 eV), the quaternary nitrogen (400.8 eV), and the oxidized nitrogen (402.9 eV), respectively. The calculated surface molar ratio of those four peaks can be compared quantitatively: yielding: pyridinic nitrogen (25.4%), pentagonal pyrrolic nitrogen (39.1%), quaternary nitrogen (32.0%), and oxidized nitrogen (3.5%), respectively. Pyridinic nitrogen, that is N bonded to two C atoms in six-membered rings at the edge of the graphitic carbon layer, would provide the main initial active sites for electrochemical behaviors^18^,^19^.

The N_2_ adsorption-desorption isotherms and pore texture properties of ACMs were further investigated. We found that all samples present characteristic type-IV sorption isotherms with saturation at a low relative pressure (P/P_0_) of ~0.1, indicating a pronounced hysteresis of the mesoporous structures (see Fig. S4)^20^. Previous hard-or soft-template methods have been used extensively to design the pore structure. However, previous studies suffered from massive use of template agents and tedious template removal processes as for either hard-template strategies or precise control of the low carbon precursor concentrations in small scale studies using soft-template strategies^21^,^22^,^23^,^24^,^25^,^26^. The mesopores developed by template methods are always two-dimensional and long-range, which result in a long transformation distance^27^. Template-free and scale-up synthetic strategies are herein introduced to manipulate the pore characteristics in 3D as well as the functional nitrogen content via control of the carbonization temperature. We note that the N_2_ adsorption quantity and the total pore volume (*V*_total_) both increase with increasing carbonization temperature (see Table 1). The average mesopore size of the samples carbonized at 600–800 °C is centered at 30 nm, while a much smaller pore size of ~3 nm is obtained by increasing the temperature to 900–1000 °C. CNTs involving ACMs have both a higher surface area (up to 613.8 m^2^/g) and a higher pore volume (up to 0.366 cm^3^/g, indicating that CNTs offer additional pores in the formation of ACMs. These behaviors might be explained as follows: before reaching the carbonization temperature, the CNTs-backboned PAN molecule transiently melts and becomes cyclized in the stabilization treatment process, so that the resulting material has a tendency to adhere strongly to the inner CNTs; then during the thermal decomposition in the carbonization process, the gas evolution (of HCN, carbon-oxides, water, ammonia, nitrogen) starts^28^,^29^, and becomes involved in the formation of mesopores and macropores^30^. This surmise is supported by the Differential Scanning Calorimeter (DSC) measurements of PAN and PANCNT monoliths in air (Fig. S5). We have also observed that the DSC profiles of PAN and PANCNT monoliths possess an exothermal character, in which the peak associated with the cyclization reaction is shifted from 286.1 °C (PAN) to a higher temperature of 323.5 °C (PANCNT). This cyclization process gives rise to the formation of a thermally stable aromatic ladder polymer^31^.

## Discussion

On the other side, the high content of nitrogen (up to 9.0% for ACMCNT-7) can improve the wettability and thus increase the surface utilization of the carbon materials in the electrolyte and thereby enhance the mass transfer efficiency^32^. As is already known for the activated carbons with nitrogen functionality, the capacitance can be enhanced by pseudo-faradaic charge transfer^33^. The PANCNT composite with high nitrogen content but with low surface area has been reported by Beguin *et al*. to make clear the pseudocapacitance effect when the composite is used as a supercapacitor electrode^31^, and the carbon atoms are next to the pyridinic nitrogen specie as has been demonstrated by Guo *et al*. to be able to create Lewis active sites that are vital for an electrochemical reaction^34^. In our case, the contribution of this effect due to the relatively low surface area will be higher than the nitrogen for the composite prepared at 900 °C (ACMCNT-7), because they have more accessible pores than those obtained at 600 °C. Such features with three-dimensional (3D) hierarchical ordered porous structures in a monolith would be beneficial to the ion diffusion and to the contact of carbon materials with the electrolyte, thus enabling their enhanced performance when used as supercapacitor electrode materials^35^,^36^.

However, due to the self-assembling characteristics of CNTs, the use of them in composites is always in bundles, which hinders their large scale applications. However, the homogeneous distribution of CNTs in the carbon/carbon composite is largely dependent on the dispersion level of CNTs in the DMSO, and the homogeneous distribution can be realized using a linear zwitterionic surfactant 3-(N, N-dimethylstearylammonio) propanesulfonate mixed with sodium cholate as the dispersant. Previous work shows that dipole/dipole electrostatic attraction was pioneeringly used as the driving force to disassemble the carbon nanotube bundles into individual tubes in aqueous solutions^37^,^38^. In this work, highly dispersed CNTs in DMSO can be prepared by carrying out a Dynamic Light Scattering (DLS) size distribution (see Fig. S6).

**Figure 5:**
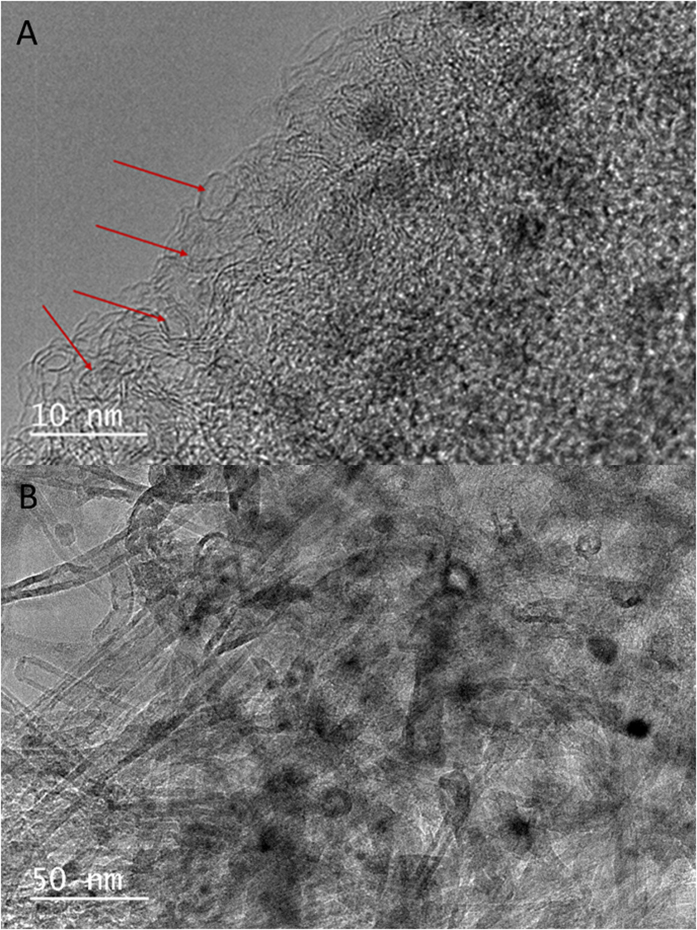
TEM images of (**A**) ACM-5 (higher magnification) and (**B**) ACMCNT-7 (lower magnification).

After carbonization, the TEM images in Fig. 5 reveal that PAN derived carbon monoliths have a mesoporous graphitic structure (pointed out by the red arrows) in higher temperatures at 900 °C (ACM-4, *S*_BET_ = 187.5 m^2^/g) and at 1000 °C (ACM-5, *S*_BET_ = 551.2 m^2^/g), while very much less mesopores with a graphitic structure were formed at temperature less than 700 °C (Fig. S7) (ACM-3, *S*_BET_ = 13.7 m^2^/g), which can be confirmed by BET analysis. The previously highly dispersed CNTs are homogenously surrounded by PAN-derived carbons in the ACM (Fig. 5B), as well as forming more numerous mesopores (ACMCNT-7, *S*_BET_ = 613.8 m^2^/g), which are connected and overlapped with each other to form a 3D porous structure on the C/C composite skeleton. This kind of porous structure is very important from an applications perspective, including electrodes for use in supercapacitors, because each electrodes can facilitate better diffusion and transport of the electrolyte.

**Figure 6:**
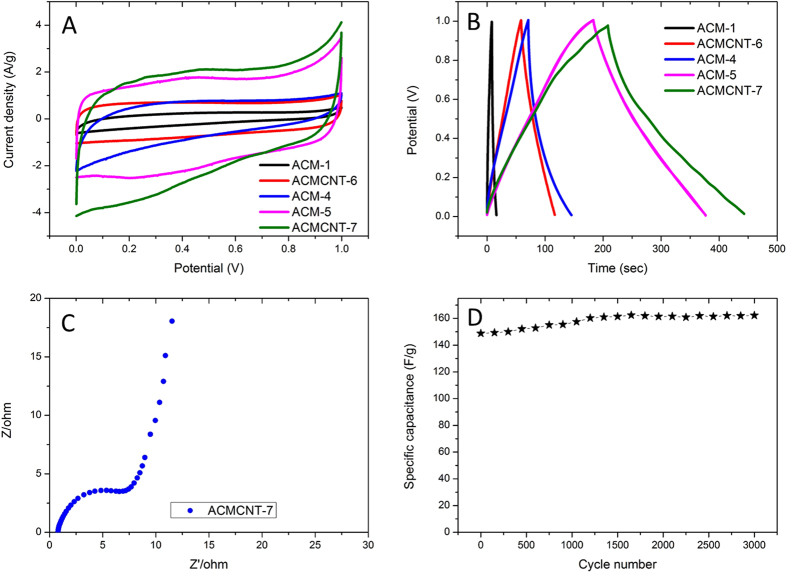
Cyclic voltammograms at a scan rate of 10 mV/s (**A**), galvanostatic charge/discharge curves (**B**), a Nyquist plots in the range of 100 kHz to 10 mHz of the as-prepared samples (**C**), and a cycle test of the supercapacitor based on ACMCNT-7, where a test was carried out at a scan rate of 50 mV/s in 1 M H_2_SO_4_ over 3000 cycles (**D**).

The ACMs were used as supercapacitor materials to probe and improve their capacitive performance and diffusion efficiency. CNTs incorporated ACMs show better electronic conductivity and we found it unnecessary to add any other conductive additives (e.g. acetylene black) to the working electrode. Figure 6A represents the CV curves of the as-prepared samples in 1 M H_2_SO_4_ taken at a scan rate of 10 mV/s. The CV curves show quasi-rectangular shapes for these ACMs, demonstrating a typical characteristic behavior of double-layer capacitances. Figures showing the specific capacitance of ACM-1, ACM-4, ACM-5, ACMCNT-6, and ACMCNT-7 can reach values of 10, 75.6, 184, 58.0, 216 F/g, respectively. Assuming that 900 °C and 120 min are the optimal carbonization temperature and time, comparison of the results from ACM-4 (75.6 F/g) and ACMCNT-7 (216 F/g) shows that a higher capacitance value is achieved by the ACMCNT-7. With further increasing the temperature, the capacitance value of ACM-5 (184 F/g) is getting smaller than that for ACMCNT-7. Hence, we can assume that due to the incorporation of CNTs, the increased additional pores as well as a higher nitrogen content (up to 9%) is required to obtain optimal performance in the electrochemical properties. Beguin *et al*. previously devised experiments to figure out the optimum proportion between CNTs and PAN, and a comparison between the values for the samples with different nitrogen content, demonstrating that high nitrogen content especially for pyridinic nitrogen gives a dominant contribution to the hybrid capacitance properties, including both the supercapacitor and pseudocapacitance behaviors^31^. Recently, Pan *et al*. designed a novel kind of CNTs/N-doped carbon polyhedra structure via using CNTs as a substrate for the *in situ* growth of metal-organic frameworks (MOFs), [Zn(2-MeIM)_2_] (2-MeIM: 2-methylimidazolate, ZIF-8) with a subsequent annealing process^39^,^40^,^41^,^42^. The hybrids demonstrate a higher specific capacitance of 308.0 F/g due to the improved degree of graphitization and the higher surface area, compared with our materials.

The galvanostatic charge/discharge curves reveal that an almost symmetrical triangle is obtained without an obvious voltage drop related to the internal resistance during the changing of polarity (see Fig. 6B), suggesting the fast transmission of ions in the hierarchical macro-mesoporous structure. An Electrochemical Impedance Spectroscopy (EIS) test was also carried out to understand the capacitive behavior of ACMCNT-7 over the frequency range of 100 kHz to 0.01 Hz. Evidence for sufficient ion diffusion is confirmed by the Nyquist plot (see Fig. 6C). The low-frequency segment is nearly perpendicular, suggesting a superior capacitive behavior. The Equivalent Series Resistance (ESR) is estimated to be 6.0 Ω by the diameter of the semicircle at the axis, indicating a good electrical conductivity. Furthermore, the CV results in Fig. 6D exhibit good cyclability, which is especially desirable for supercapacitors, thereby increasing the capacitance value by ~8% after 3000 cycles at a scan rate of 50 mV/s (Fig. 6D). The capacitance of the ACMs does not change significantly (remains at ~65%) with the increasing scan rates, giving a constant value (135 F/g), even beyond a high scan rate of 200 mV/s (Fig. S8).

In conclusion, for the first time, three-dimensional nitrogen-doped porous activated carbon monoliths (3D-NDP-ACMs) with controllable macro-mesopores and regulative morphologies prepared by a two-step template-free method have been demonstrated to have high capacitance properties as electrode materials. CNTs offer additional pores through the formation of 3D-NDP-ACMs by the shrinkage of the outer PAN layer that tends to adhere strongly to the inner CNTs layer during thermal decomposition. Such features obtained with 3D hierarchical ordered porous structures in their monoliths and the high wettability of the nitrogen functionalities would increase the surface utilization of the carbon materials and be beneficial to the ion diffusion in the electrolyte. The remarkable capacitance properties of this kind of new bulk material are attributed to the synergistic effects between the conductive backboned-CNTs and the pseudocapacitance behaviors of PAN-derived nitrogen functionalities. These methods are here shown to be general and scalable for the production of a new class of activated carbon monolith materials derived from polymers, including polyimide, polyvinyl alcohol, cellulose, etc. that are under study in our research groups.

## Methods

### Materials

Polyacrylonitrile (PAN, MW 150,000) was purchased from Sigma-Aldrich and was used without further purification. NANOCYL^TM^ NC7000 series MWCNTs (9.5 nm in diameter and 1.5 μm in length) was manufactured by Nanocyl. Other chemicals (unless noted) came from Wako Pure Chemical Industries, Ltd., or Sigma–Aldrich, Inc., Japan.

### Materials synthesis

MWCNT mono-dispersions (0.65 wt. %) were prepared by modification of MWCNT bundles in dimethyl sulfoxide (DMSO) with a sulfonate/quaternary-ammonium type of zwitterionic surfactants according to the previous reports by our group^37^,^38^. PAN (0.8 g) was dissolved into DMSO (8.75 mL) and H_2_O (1.25 mL) under stirring at 120 °C. After becoming completely dissolved, the PAN translucent solution was then transferred into a kind of glass vessel and kept for 40 min for the occurrence of the phase separation. To remove the DMSO and the unwanted surfactants in the as-prepared PAN monoliths, the monoliths were immersed in methanol under mild shaking for 24 h. Finally, the monoliths were dried under vacuum for 48 hours. For consistency, all PAN monolith samples reported in this study were prepared by the same procedure. In the case of the PANCNT monoliths, PAN (0.8 g) was dissolved into the CNTs/DMSO (8.75 mL) and H_2_O (1.25 mL) solutions under stirring at 120 °C. The following procedures for the PANCNT monoliths are identical to those for the PAN monoliths. The as-prepared PAN and PANCNT monoliths were firstly subjected to oxidative annealing by heating in a chamber furnace in air to a temperature of 220 °C at a rate of 5 °C/min, before being held at that temperature for 1 h. After naturally cooling to room temperature, the stabilized PAN (denoted as PAN-P) was then pyrolysed by heating under a mixed Ar/CO_2_ (3:1) atmosphere to a temperature 600 °C, 700 °C, 800 °C, 900 °C, 1000 °C, respectively, at a rate of 5 °C/min, before being held at their respective temperatures for 1 h or 2 h. The hierarchical porous carbon monoliths held at their corresponding temperatures (600 °C, 700 °C, 800 °C, 900 °C, and 1000 °C) for 2 h were denoted as ACM-1, ACM-2, ACM-3, ACM-4 and ACM-5, correspondingly. The ACMCNT-6 and ACMCNT-7 monoliths were prepared at the temperatures of 600 °C and 900 °C for 2 h, respectively. The ACM-8, ACM-9, ACMCNT-10 and ACMCNT-11 monoliths were prepared at 600 °C, 900 °C, 600 °C and 900 °C, respectively, with a hold time of 1 h.

### Characterization

Specific surface areas and mesopore size distributions were measured using nitrogen sorption at 77.3 K and using a 3Flex volumetric adsorption analyzer (Micromeritics Instrument Corporation, USA). Prior to the measurement, the samples were degassed at 60 °C for the PAN monoliths and 300 °C for 6 h under vacuum. The specific surface areas were determined according to the Brunauer-Emmett-Teller (BET) method in the relative pressure range of 0.05–0.3. The pore size distribution (PSD) of the samples was calculated using the Barrett-Joyner-Halenda (BJH) method. The surface chemical composition of the samples was studied by X-ray photoelectron spectroscopy (XPS) on a PHI 5000 Versa Probe (ULVAC-PHI, Inc.), using Al Kα radiation (1486.6 eV). The operating pressure in the analysis chamber was maintained below 1.0 × 10^−9^ Torr. Wide scan spectra in the binding energy range of 1400-0 eV were recorded in a step energy of 1.0 eV with a pass energy of 100 eV. High resolution spectra of the elemental signals were recorded in a step energy of 0.2 eV with a pass energy of 23.5 eV. Both measurements were conducted with a focus size, power and voltage of the X-ray beam for 100 μm, 25 W and 15 kV. The morphology and structure of the samples were analyzed using a Low Damage Scanning Electron Microscope JSM-7500FA with an accelerating voltage of 1.0 eV and an emission current of 10 μA. The porous structure of the samples was analyzed by HRTEM (JEM-ARM200F). Raman spectra were measured *via* a Renishaw inVia Raman microscope fitted with a 532 nm laser, and calibrated against a silicon wafer reference. The sheet resistance of the prepared monoliths were tested by a Surface Resistance Meter (Loresta EP, Model MCP-T360, Mitsubishi Chemical Co., Japan) at room temperature. The thermal behaviors of the samples were performed using a differential scanning calorimeter (Model DSC22, SII Nano Technology Inc.) connected to a thermal analysis system (model, SSC 5100).

### Electrochemical measurements

The electrochemical performance of the samples was measured by cyclic voltammetry (CV) at room temperature in a three-electrode cell using a Model-600C electrochemical analyzer (BAS Inc., Tokyo, Japan). The working electrode was fabricated by casting the obtained carbon material of 2 mg/mL onto a vitreous glassy carbon disk electrode. An Ag/AgCl (3.0 M NaCl) electrode and a Pt wire were used as a reference electrode and a counter electrode, respectively. The CV curves were obtained at various scan rates in a 1.0 M H_2_SO_4_ electrolyte. Electrochemical impedance spectroscopy (EIS) of the samples was measured in a 1.0 M H_2_SO_4_ solution using a sinusoidal signal amplitude of 5 mV over the frequency range from 10^5^ Hz to 10^−2^ Hz at an open circuit potential. All measurements were carried out at room temperature. The average specific capacitance of the samples was calculated from the cyclic voltammograms obtained, using the following equation:


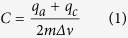


where *q*_a_ and *q*_c_ are the integrated anodic and cathodic voltammetric charges, respectively; *m* and *Δν*, respectively, refer to the mass of the electro-active material and the potential window of the cyclic voltammograms, respectively.

## Additional Information

**How to cite this article**: Wang, Y. *et al*. Nitrogen-doped porous carbon monoliths from polyacrylonitrile (PAN) and carbon nanotubes as electrodes for supercapacitors. *Sci. Rep.* **7**, 40259; doi: 10.1038/srep40259 (2017).

**Publisher's note:** Springer Nature remains neutral with regard to jurisdictional claims in published maps and institutional affiliations.

## Supplementary Material

Supplementary Information

## Figures and Tables

**Table 1:** Specific surface areas, pore characteristics and C, N and O contents of the samples.

Sample	S_BET_^*a*^ (m^2^/g)	V_micro_(cm^3^/g)	V_macro-meso_(cm^3^/g)	V_total_(cm_3_/g)	D^*b*^/nm	C^*c*^	N^*c*^	O^*c*^
PAN	235.7	0.005	1.277	1.282	11.2	78.1	21.9	0
ACM-1	4.7	0.002	0.024	0.026	27.2	79.2	17.2	3.6
ACM-2	8.1	0.004	0.028	0.032	26.1	82.5	12.8	4.7
ACM-3	13.7	0.005	0.020	0.025	33.3	84.6	12.4	3.0
ACM-4	187.5	0.067	0.103	0.170	3.08	88.4	9.2	2.4
ACM-5	551.2	0.217	0.100	0.317	2.93	93.7	3.1	3.2
ACMCNT-6	8.5	0.005	0.006	0.011	16.4	81.0	15.8	3.2
ACMCNT-7	613.8	0.237	0.129	0.366	3.09	84.7	9.0	6.3

^*a*^Derived from BET. D^*b*^ refers to BJH adsorption average pore diameter, derived from BET. C^*c*^, N^*c*^, O^*c*^ refer to weight percentage, derived from XPS. BET = Brunauer, Emmett and Teller, BJH = Barrett-Joyner-Halenda and XPS = X-ray Photoelectron Spectroscopy.
