# Corrigendum: Nitrogen-doped porous carbon monoliths from polyacrylonitrile (PAN) and carbon nanotubes as electrodes for supercapacitors

**DOI:** 10.1038/srep46889

**Published:** 2017-08-24

**Authors:** Yanqing Wang, Bunshi Fugetsu, Zhipeng Wang, Wei Gong, Ichiro Sakata, Shingo Morimoto, Yoshio Hashimoto, Morinobu Endo, Mildred Dresselhaus, Mauricio Terrones

Scientific Reports 7: Article number: 40259; 10.1038/srep40259 published online: 01 11 2017; updated: 08 24 2017.

In the Supplementary Information file originally published with this Article, References 1–5 were omitted. This error has been corrected in the Supplementary Information that now accompanies the Article.

