# Supplementary material for: Nitrogen-doped porous carbon monoliths from polyacrylonitrile (PAN) and carbon nanotubes as electrodes for supercapacitors
Source: Sci Rep. 2017 Jan 11;7:40259. doi: 10.1038/srep40259 (PMC5225489; doi:10.1038/srep40259)
Supplement: Supplementary Information [file srep40259-s1.docx]

SUPPORTING INFORMATION FOR

**Nitrogen-doped porous carbon monoliths from polyacrylonitrile (PAN) and carbon nanotubes as electrodes for supercapacitors**

Yanqing Wang^1^^*^, Bunshi Fugetsu^1, 2^, Zhipeng Wang^3^, Wei Gong^1^, Ichiro Sakata^1, 2^, Shingo Morimoto^3^, Yoshio Hashimoto^3^, Morinobu Endo^3^, Mildred Dresselhaus^4^

& Mauricio Terrones^5^

^1^School of Engineering, The University of Tokyo, Bunkyo-ku, Tokyo 113-0032, Japan.

^2^Policy Alternative Research Institute, The University of Tokyo, Bunkyo-ku, Tokyo 113-0032, Japan.

^3^Institute of Carbon Science and Technology, Shinshu University; 4-17-1 Wakasato, Nagano 380-8553, Japan.

^4^Research Laboratory of Electronics, Department of Electrical Engineering and Computer Science, Department of Physics, Massachusetts Institute of Technology, 77 Massachusetts Avenue, Cambridge, MA 02139-4307, USA.

^5^Department of Physics, Department of Chemistry, Department of Materials Science and Engineering and Center for 2-Dimensional and Layered Materials, The Pennsylvania State University, University Park, PA 16802, USA.

**
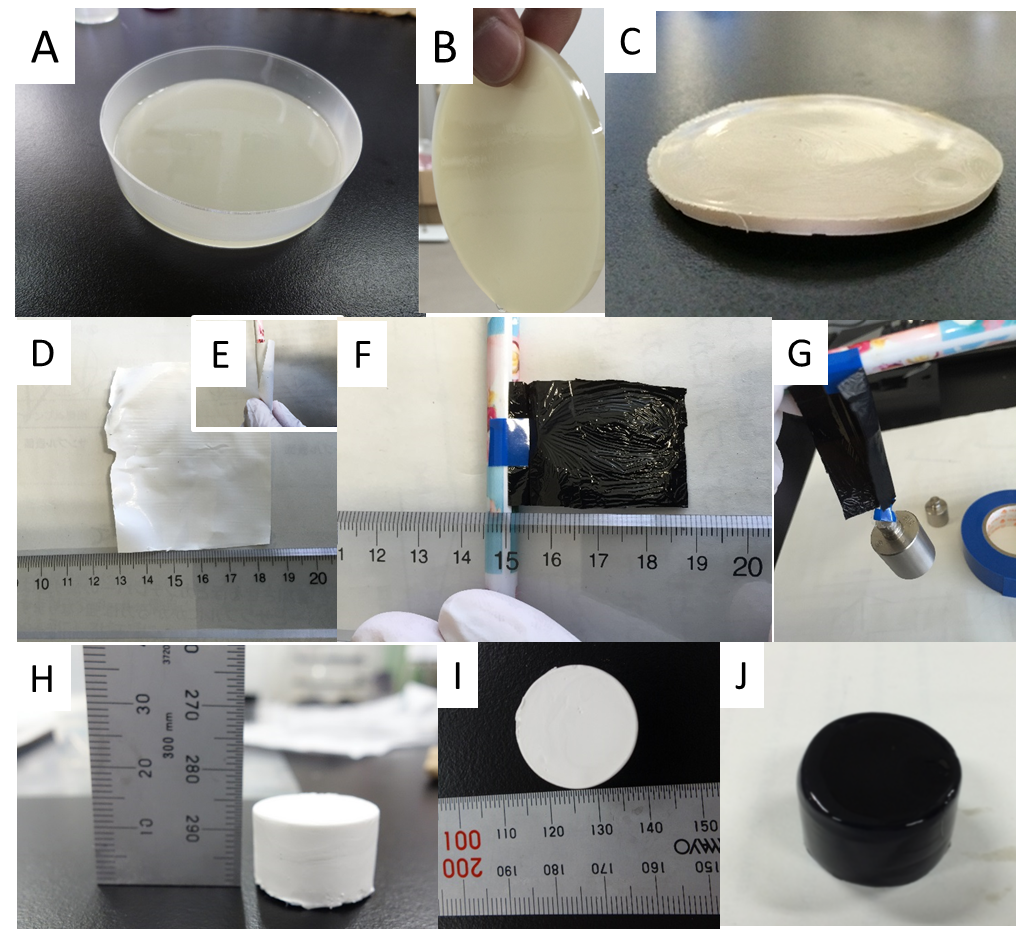
**

Fig. S1. Photos of plate-like PAN before phase separation (A), after phase separation (B), as-prepared plate-like PAN after vacuum drying (C), and photos of PAN thin film (D), rolled-up PAN film (E) and PANCNT film (F), robust PANCNT film pulled with a 50g suspended weight (G), and photos of 3D hierarchical PAN monoliths (H and I), and of a PANCNT monolith (J).

**
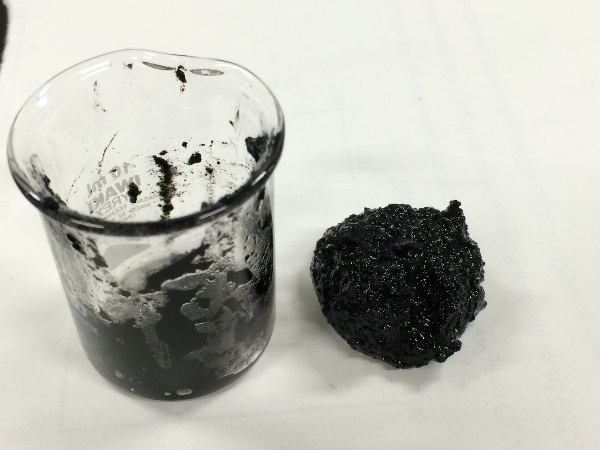
**

Fig. S2. Photo of a PANCNT composite with high CNTs concentrations (10wt. %) after phase separation.

**
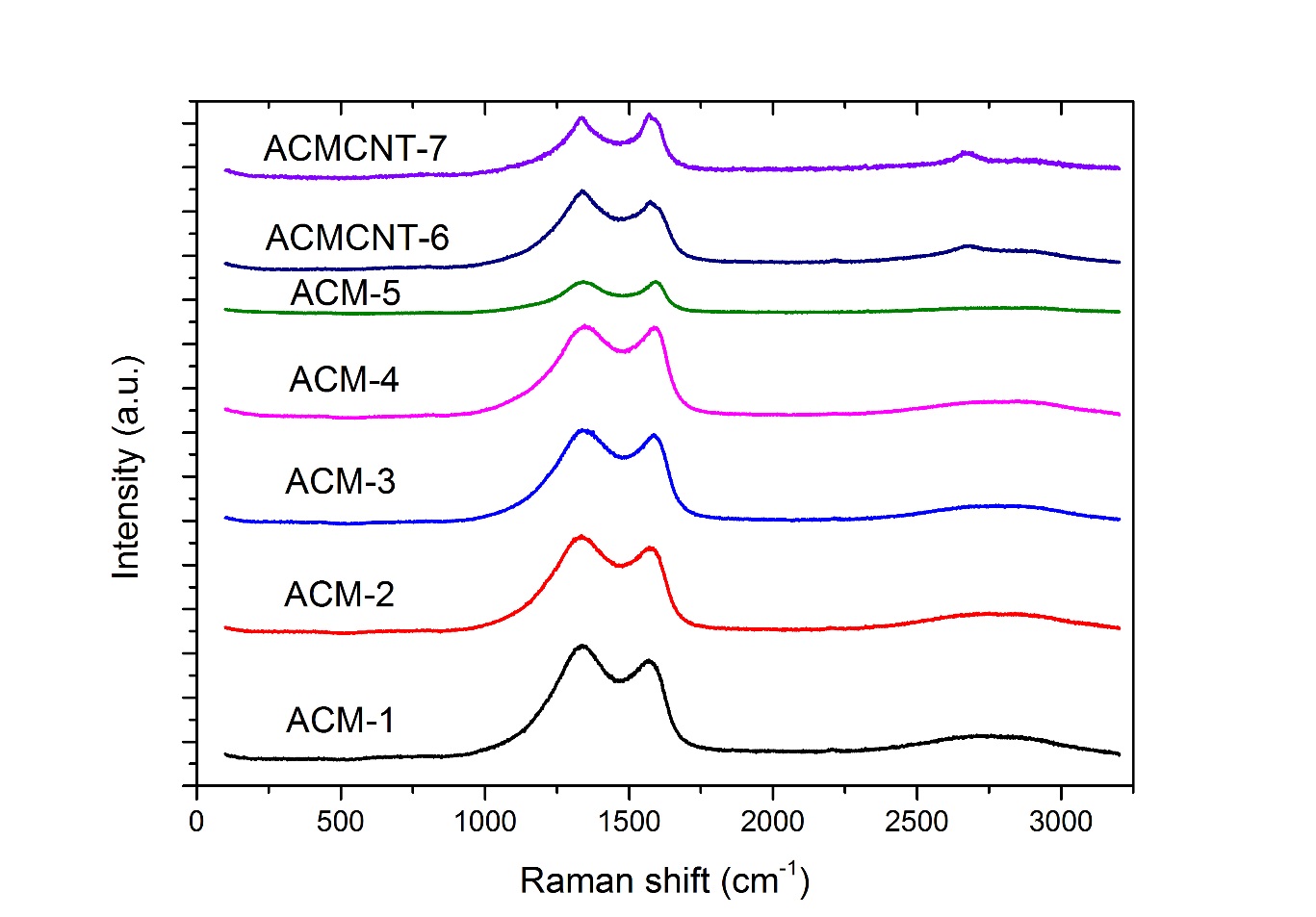
**

Fig. S3. Raman spectra of the various activated carbon monolith (ACM) samples described in the text.

Table S1 Raman spectra frequency and relative intensity parameters of the as-obtained ACMs, where the ratios of the peak intensities were calculated by the areas under the lines of the peaks.

| Sample | D band  (cm^-1^) | G band  (cm^-1^) | 2D band (cm^-1^) | *I_D_*/*I_G_* | *I_2D_*/*I_G_* |
| --- | --- | --- | --- | --- | --- |
| ACM-1 | 1341.5 | 1570.6 | 2739.9 | 3.91 | 1.97 |
| ACM-2 | 1344.6 | 1574.5 | ------ | 3.46 | ------ |
| ACM-3 | 1354.5 | 1583.5 | 2797.8 | 3.54 | 3.02 |
| ACM-4 | 1359.4 | 1584.9 | 2818.5 | 3.32 | 3.40 |
| ACM-5 | 1348.1 | 1586.4 | 2928.5 | 3.36 | 1.73 |
| ACMCNT-6 | 1343.8 | 1577.1 | 2738.2 | 2.69 | 1.76 |
| ACMCNT-7 | 1342.8 | 1574.9 | 2707.7 | 2.38 | 1.50 |

Table S2 Contents of C and N in the N-doped ACMs

| Sample | Temperature  (K) | Hold time (h) | C (wt. %) | N (wt. %) | Electrical conductivity (Ω/sq) |
| --- | --- | --- | --- | --- | --- |
| PAN-R | — | — | 78.1 | 21.9 | — |
| ACM-1 | 873 | 2 | 79.2 | 17.2 | 396.3x10^3^ |
| ACM-2 | 973 | 2 | 82.5 | 12.8 | 1.655x10^3^ |
| ACM-3 | 1073 | 2 | 84.6 | 12.4 | 72.9 |
| ACM-4 | 1173 | 2 | 88.4 | 9.2 | 33.7 |
| ACM-5 | 1273 | 2 | 93.7 | 3.1 | 13.4 |
| ACMCNT-6 | 873 | 2 | 81.0 | 15.8 | 4.67 x10^3^ |
| ACMCNT-7 | 1173 | 2 | 84.7 | 9.0 | 6.3 |
| ACM-8 | 873 | 1 | 79.0 | 18.3 | 427.1x10^3^ |
| ACM-9 | 1173 | 1 | 87.1 | 10.8 | 396.3 x10^3^ |
| ACMCNT-10 | 873 | 1 | 81.1 | 16.5 | 9.2 x10^3^ |
| ACMCNT-11 | 1173 | 1 | 84.3 | 10.9 | 25.9 |


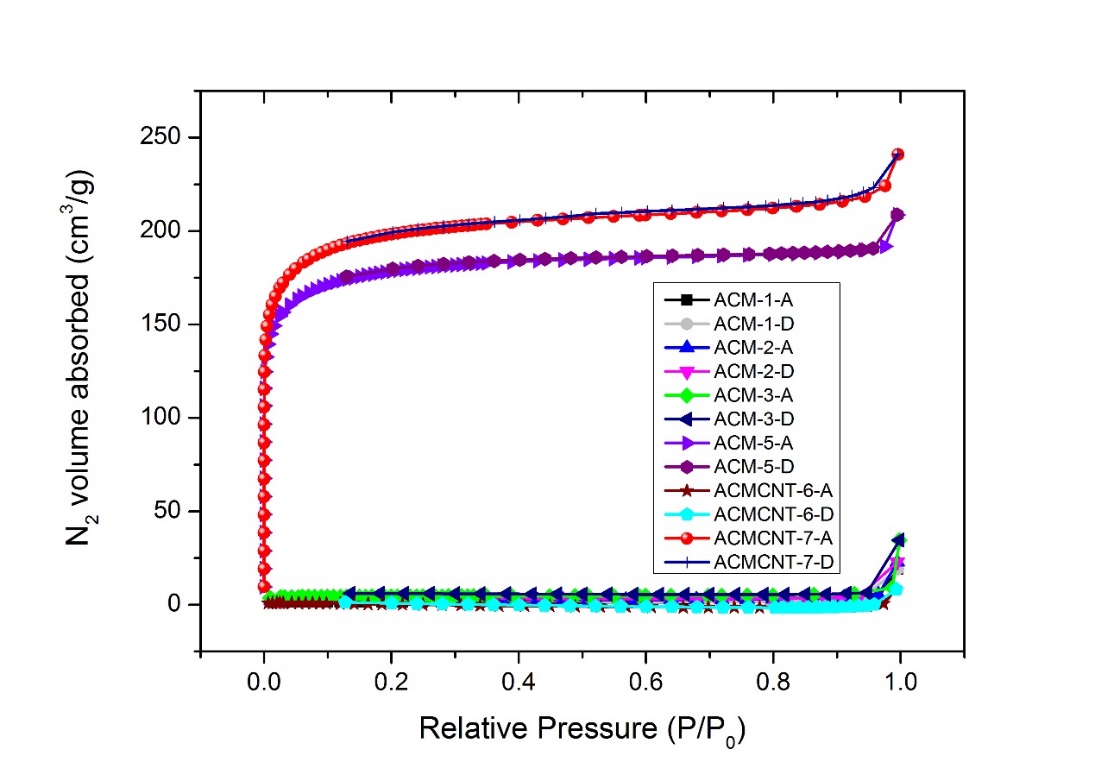


Fig. S4. Nitrogen sorption isotherms of the carbon monoliths outlined in this work: A (adsorption) and D (desorption), where ACM denotes the activated carbon monolith, ACMCNT denotes the activated carbon monolith that also contain carbon nanotubes.


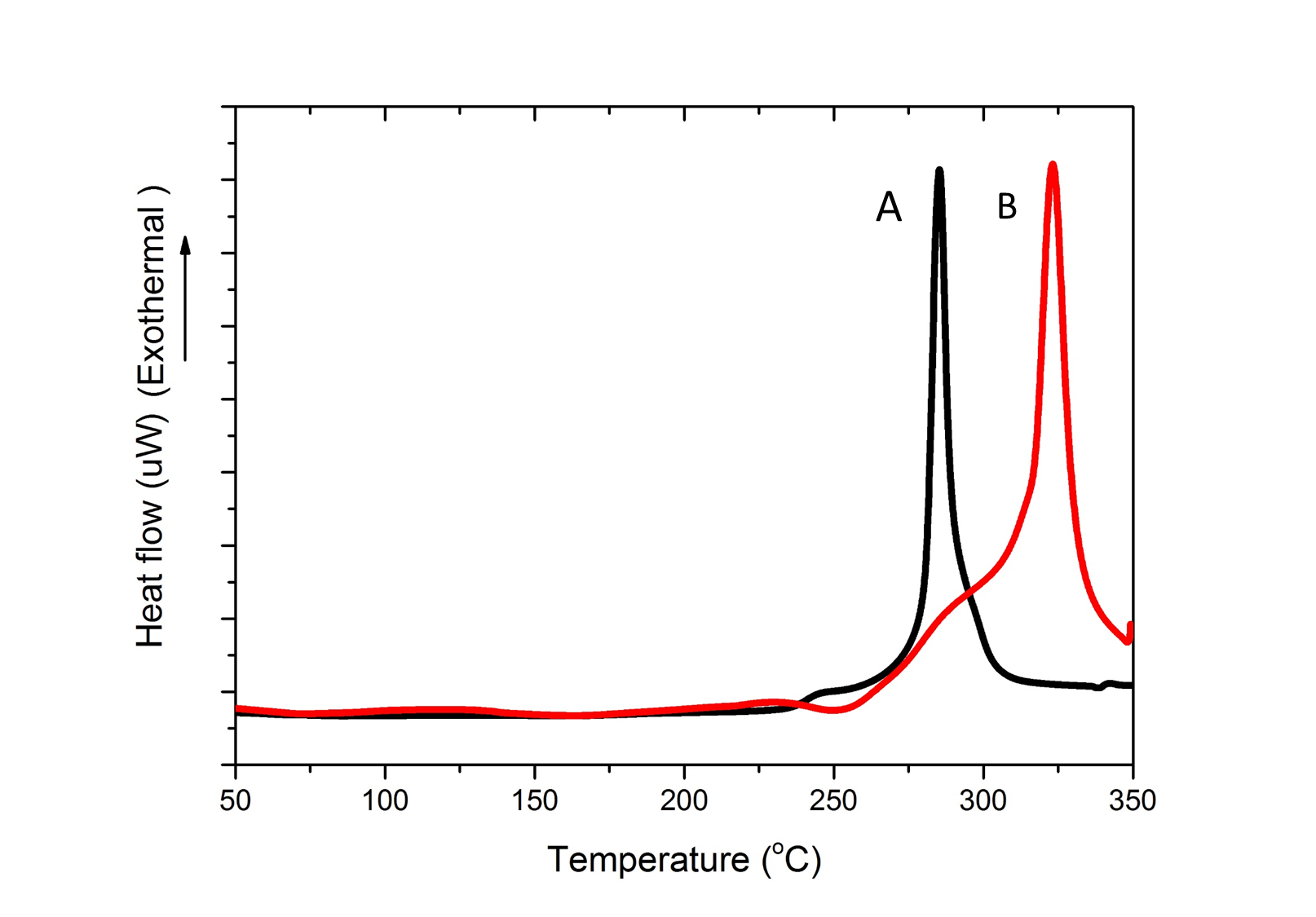


Fig. S5. Differential Scanning Calorimeter (DSC) curves of PAN (A) and PANCNT (B) monoliths during stabilization treatments in air.


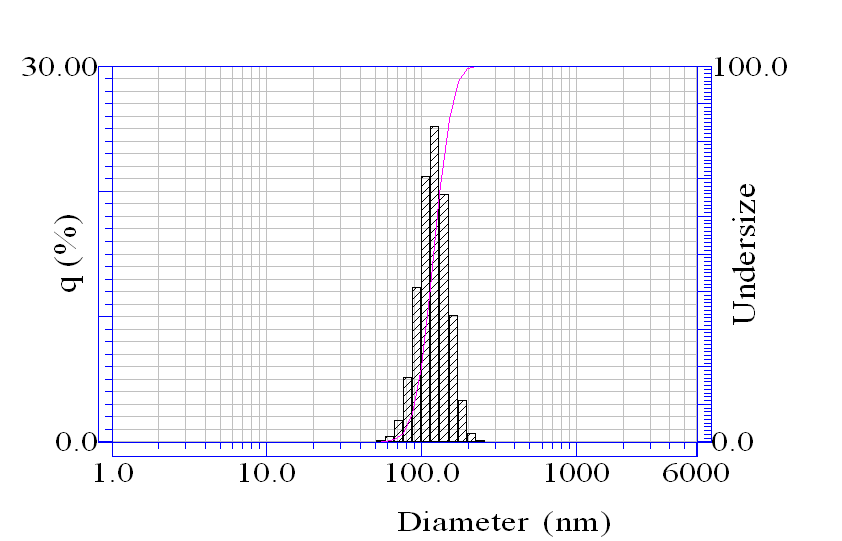


Fig. S6. Dynamic light scattering of the aqueous CNT solution in DMSO. Note: “q%” refers to “cumulative particle size %” and “Undersize” refers to “cumulative percent passing through the light pulse”.


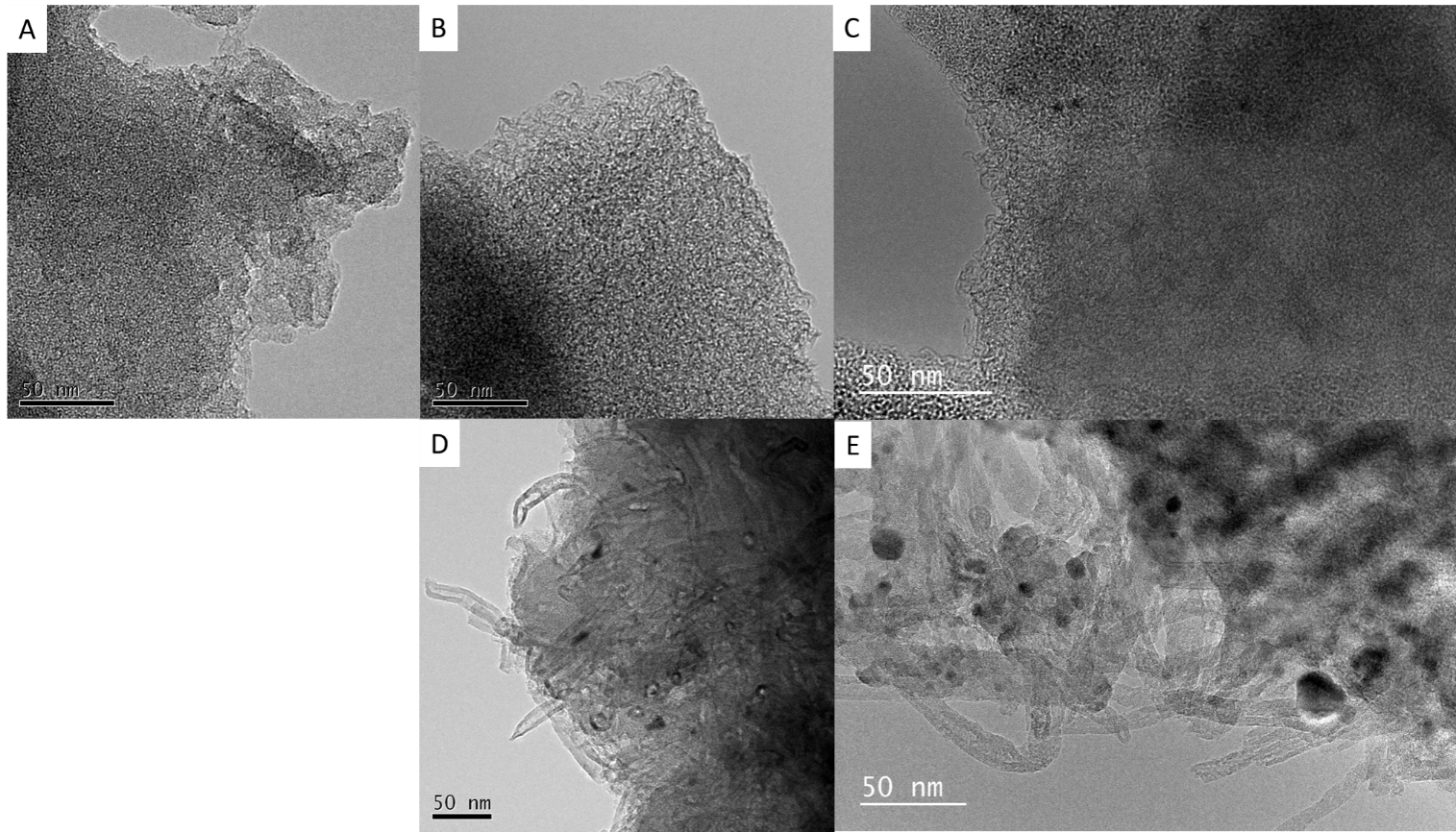


Fig. S7. TEM images of ACM-2 carbonized at 700 °C (A), ACM-4 carbonized at 900 °C (B), ACM-5 carbonized at 1000 °C (C), and ACMCNT-6 carbonized at 600 °C (D), ACMCNT-7 carbonized at 900 °C (E). Note: all the scale bars are 50 nm.


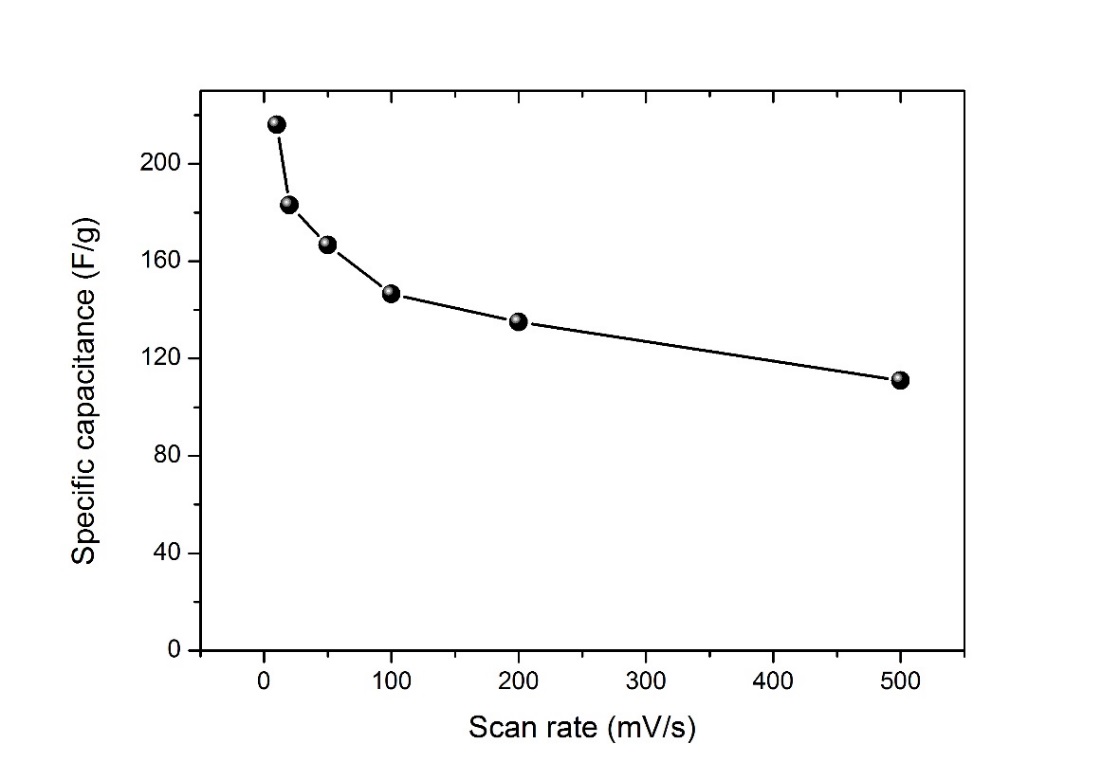


Fig. S8. Specific capacitance of ACMCNT-7 measurements taken over various scan rates (10 mV/s, 20 mV/s, 50 mV/s, 100 mV/s, 200 mV/s and 500 mV/s) in 1M H_2_SO_4_, and capacitance retention values of 85% (20 mV/s), 77% (50 mV/s), 68% (100 mV/s), 65% (200 mV/s) and 52% (500 mV/s) were obtained.
